# Epoprostenol as an alternative anticoagulant for pediatric aquadex therapy: a safe and feasible option

**DOI:** 10.3389/fped.2026.1858921

**Published:** 2026-07-22

**Authors:** Buria Naeem, Jenna Harper, Coleen McSteen, Dana Y. Fuhrman

**Affiliations:** 1 Department of Pediatric Critical Care Medicine, Le Bonheur Children's Hospital, University of Tennessee, Memphis, TN, United States; 2 Department of Critical Care Medicine, Division of Pediatric Critical Care Medicine, UPMC Children's Hospital of Pittsburgh, Pittsburgh, PA, United States; 3 Division of Critical Care Medicine, Cincinnati Children's Hospital Medical Center, Cincinnati, OH, United States; 4 Division of Nephrology and Hypertension, Cincinnati Children's Hospital Medical Center, Cincinnati, OH, United States

**Keywords:** acute kidney injury, aquadex, continuous renal replacement therapy, epoprostenol, fluid removal, prostacyclin

## Abstract

**Background:**

The Aquadex ultrafiltration system offers a low extracorporeal volume option for the pediatric population. To maintain circuit patency, it relies on conventional anticoagulation, and data on alternative anticoagulation strategies are limited. Epoprostenol, a prostacyclin analog that inhibits platelet aggregation, may provide a safe alternative. The objective of this study was to describe the use of epoprostenol as the sole anticoagulant for Aquadex ultrafiltration therapy in critically ill children and to evaluate its safety, feasibility, and impact on circuit patency.

**Methods:**

We conducted a single-center retrospective study of children (<18 years) who received Aquadex therapy with epoprostenol anticoagulation between September 2022 and September 2025 at UPMC Children's Hospital of Pittsburgh. Safety and efficacy were assessed by platelet count, bleeding events, vasoactive-inotropic scores (VIS), and circuit performance.

**Results:**

Forty-six patients underwent 52 treatments. Median (IQR) age was 35.5 (12.5–114) months. Primary indications were fluid overload (88.5%) and acute kidney injury (11.5%). The median therapy duration was 3 [2–9] days, with a filter-to-day ratio of 0.5 (0.3–0.5). Platelet counts rose from a median (IQR) of 113 (×10⁹/L) prior to therapy to 126 (×10⁹/L) at 72 h, with a median of 0 [0–1] platelet transfusions per day. The median VIS was 4 [0–12] prior to therapy and 0 [0–8] at 48 h; vasoactive-inotropic requirements did not increase at any time point. No bleeding events directly attributable to epoprostenol or therapy discontinuations due to hypotension occurred. Circuit patency was maintained, with a median of 0 (0–1) interruptions per course due to clotting or clogging.

**Conclusions:**

Epoprostenol appears to be a feasible anticoagulant for pediatric Aquadex therapy, with preserved circuit function, no increase in vasoactive-inotropic requirements, and no observed bleeding events. These findings support its further evaluation as an alternative anticoagulant for pediatric Aquadex Therapy.

## Introduction

Despite significant advances in critical care medicine, fluid overload remains a major cause of pediatric morbidity. Children in the intensive care unit (ICU) frequently develop fluid overload, which is associated with longer duration on mechanical ventilation, increased hospital stay, and worse renal outcomes. While diuretic therapy is used as first-line therapy to manage fluid overload, extracorporeal modalities such as hemodialysis, continuous renal replacement therapy (CRRT), and slow continuous ultrafiltration (SCUF) are frequently needed to manage volume status (1–3).

The Aquadex Flex Flow system (CHF Solutions Inc., Prairie, MN), originally designed for diuretic-resistant adult heart failure, has been investigated in the pediatric population due to its low extracorporeal volume and suitability for small patients. Studies have demonstrated its feasibility and safety for a range of kidney support modalities in children, including SCUF and continuous veno-venous hemofiltration (CVVH) (1, 4). As with all extracorporeal therapies, maintaining circuit patency is crucial, and anticoagulation is required to prevent clotting. Unfractionated heparin remains the standard anticoagulant used with Aquadex, but can be limited by bleeding risk and heparin-induced thrombocytopenia, and is usually contraindicated in patients with coagulopathy or liver disease (5–7). Citrate-based anticoagulation can be a reasonable alternative, but it can be associated with metabolic complications (8). Therefore, in patients with liver disease or high bleeding risk, alternative agents need to be explored.

Epoprostenol is a synthetic prostacyclin analog that inhibits platelet aggregation. It has been used as a potential alternative anticoagulant in CRRT circuits, particularly in patients with liver disease, thrombocytopenia, or a high risk of bleeding (9, 10). Recent studies demonstrate that epoprostenol can provide acceptable filter life and complication rates comparable to heparin and citrate. This study aims to describe the first use of epoprostenol as an anticoagulant for Aquadex therapy in children and to evaluate its safety, feasibility, and impact on circuit patency and hemodynamic stability.

## Methods

### Study subjects

We performed a single-institution retrospective study using our institutional data at the University of Pittsburgh Children's Hospital, a free-standing quaternary pediatric referral center. Patients aged less than 18 years, between September 2022 and September 2025, in whom epoprostenol was used for anticoagulation with Aquadex were included in this study. Patients were eligible if they (i) were less than 18 years of age, (ii) underwent Aquadex therapy during the study period, and (iii) received epoprostenol as the sole anticoagulant. Beginning in September 2022, our institution adopted epoprostenol as the standard anticoagulant for all pediatric patients receiving Aquadex therapy, replacing prior protocols based on unfractionated heparin. During the study period, every consecutive pediatric patient who underwent Aquadex therapy received epoprostenol as the sole anticoagulant per this protocol. The study was approved by the University of Pittsburgh Institutional Review Board (IRB study number 21020042). A waiver of informed consent was granted, given the retrospective study design and the use of de-identified data. The study was conducted in accordance with the Declaration of Helsinki and applicable institutional and regulatory requirements.

### Study variables

Data on patient demographics, ICU location, primary reason for admission, and serum creatinine within 6 h prior to starting therapy were collected. In addition, mechanical ventilation status (defined as requiring mechanical ventilation at any point during Aquadex therapy), fluid accumulation as a percentage of total body weight prior to the initiation of Aquadex, and the Pediatric Logistic Organ Dysfunction-2 (PELOD-2) score at the time of Aquadex initiation were obtained (11). Data describing the therapy included the primary indication for Aquadex use (i.e., fluid overload or acute kidney injury), mode of therapy [slow continuous ultrafiltration (SCUF) or CVVH], vascular access site, catheter size, and blood flow rate.

Safety was evaluated by characterizing platelet count, platelet transfusions, and vasoactive requirement. We evaluated changes in platelet count on the first 3 days of therapy. The vasoactive requirement was quantified using the vasoactive-inotropic score (VIS), which was defined as the maximum vasoactive support required before starting Aquadex and at 3, 12, and 48 h after initiation. Clinically significant bleeding was defined as any bleeding event documented during Aquadex therapy that (i) required transfusion of packed red blood cells, fresh frozen plasma, or platelets specifically for hemorrhage, (ii) prompted an unplanned procedural intervention, or (iii) resulted in hemodynamic instability requiring escalation of support. Bleeding was further classified by site as gastrointestinal, pulmonary, intracranial, nasal (epistaxis), urinary (hematuria), or surgical-site. To assess circuit performance, we recorded: (i) the number of therapy interruptions per course, defined as any unplanned cessation of ultrafiltration lasting >30 min attributable to circuit clotting or clogging; (ii) the filter-to-day ratio, defined as the total number of filters used divided by the total days of therapy for that course, with lower values indicating better circuit longevity (e.g., a ratio of 0.5 corresponds to one filter remaining functional for two days); and (iii) the cause of any therapy discontinuation. A treatment course was defined as a continuous period of Aquadex therapy with no more than 24 h of interruption between sessions; any gap exceeding 24 h was considered the start of a new course.

### Aquapheresis description

Aquapheresis was performed using the Aquadex Smart Flow System (Nuwellis, Minneapolis, MN). Therapy was initiated in the intensive care unit under the supervision of the critical care and nephrology teams. The ultrafiltration rate was prescribed by the clinical team and titrated based on continuous hematocrit monitoring, hemodynamic stability, urine output, and daily fluid balance targets. Epoprostenol was infused pre-filter via the pigtail on the withdrawal (blue) limb of the extracorporeal circuit using a dedicated infusion pump. The initial infusion rate was 2 ng/kg/min, titrated to maintain adequate circuit patency and prevent filter clotting as determined by visual inspection of the filter and trending increases in filter resistance. Dose adjustments were made in 1–2 ng/kg/min increments at intervals of no less than 2 h, up to a maximum institutional dose of 10 ng/kg/min. Up-titration was triggered by a rise in transmembrane pressure or by progressive darkening of the hemofilter fibers on visual inspection. Activated partial thromboplastin time and activated clotting time were not used to titrate epoprostenol, consistent with its mechanism as a platelet aggregation inhibitor without a measurable effect on plasma coagulation assays. Epoprostenol was prepared and administered per institutional pharmacy protocol.

Continuous monitoring of pressures (withdrawal, infusion, and ultrafiltrate) was used to assess circuit integrity. In patients <15 kg, therapy was modified with pre-filter replacement fluids to provide convective clearance if needed, as previously described (1). Circuit resistance >1.5 or evidence of clot formation prompted temporary cessation of ultrafiltration and either circuit flushing or re-priming. All patients were closely monitored for hemodynamic instability, with adjustments to the ultrafiltration rate made as needed. Therapy was discontinued after 72 h, as per the circuit specifications.

### Statistical analysis

The Shapiro–Wilk test was used to assess the normality of continuous variables. Because the data were not normally distributed, continuous variables are reported as medians [interquartile ranges (IQR)] and categorical variables as counts (percentages). To evaluate safety related to anticoagulation with epoprostenol, within-patient changes in platelet count and VIS scores were compared using the Wilcoxon signed-rank test. For patients who underwent more than one treatment course, each course was treated as an independent observation in descriptive analyses. All tests were two-sided, and *p*-values ≤ 0.05 were considered statistically significant. Analyses were performed using STATA 16.1 (StataCorp, College Station, TX, USA).

## Results

### Baseline characteristics

A total of 46 patients underwent 52 Aquadex therapy courses during the study period. 42 patients (91.3%) received a single treatment course, 3 patients received 2 treatment courses each, and 1 patient received 4 treatment courses. The median (IQR) age at admission was 35.5 (12.5–114) months, and 24 (52.2%) patients were female (Table 1). The median (IQR) body surface area was 0.64 (0.4–1.11) m^2^, and the median (IQR) serum creatinine prior to therapy was 1.32 (0.49–2.83) mg/dL. The median (IQR) fluid accumulation was 9.3% (5%–12.8%) of total body weight.

**Table 1:** Patient characteristics.

Characteristic (*n* = 46)	n (%) or median (interquartile range)
Age at admission, months	35.5 (12.5–114)
Sex (female)	24 (52.1)
Race	
Black	11 (23.9)
White	32 (69.6)
Other	3 (6.5)
Body surface area at admission, m^2^	0.64 (0.4–1.11)
Creatinine prior to therapy, mg/dL	1.32 (0.49–2.83)
Fluid accumulation as a percentage of total body weight (%)	9.3 (5–12.8)
Primary Reason for Admission	
Cardiac	13 (28.3)
Endocrine	1 (2.2)
Hematologic/Oncologic	4 (8.7)
Hepatology	4 (8.7)
Renal	15 (32.6)
Sepsis	9 (19.6)
ICU location	
Cardiac	13 (28.3)
Pediatric	30 (65.2)
Neonatal	3 (6.5)
PELOD-2 at Aquadex start	5 (3–9)
Mechanically ventilated	33 (63.5)

ICU, intensive care unit; PELOD, Pediatric Logistic Organ Dysfunction.

The most common primary reasons for admission were renal (32.6%), cardiac (28.3%), and sepsis (19.6%). The majority of patients were admitted to the pediatric ICU (65.2%), followed by the cardiac ICU (28.3%) and neonatal ICU (6.5%). The median (IQR) PELOD-2 score at Aquadex initiation was 5 (3–9), and 33 (63.5%) patients were mechanically ventilated at the start of therapy.

### Aquadex therapy

The primary indication for Aquadex therapy was fluid overload (88.5%), followed by acute kidney injury (11.5%) (Table 2). The majority of treatments were performed using slow continuous ultrafiltration (SCUF; 76.9%), while CVVH was used in 23.1%. Given the small number of CVVH courses (*n* = 12) and the absence of bleeding events in either group, formal between-mode comparison was not undertaken. The most frequent vascular access site was the right internal jugular vein (61.6%), followed by the left internal jugular (9.6%) and left femoral (9.6%) veins. Catheter sizes ranged from 5 Fr to 13 Fr, with 7.5 Fr (17.3%) and 8 Fr (15.4%) being the most common. The median (IQR) blood flow rate was 2.1 (1.1–4.1) mL/kg/min.

**Table 2:** Aquadex therapy.

Characteristic (*n* = 52 Treatments)	n (%) or median (interquartile range)
Primary Indication for Therapy	
Fluid overload	46 (88.5)
Acute kidney injury	6 (11.5)
Aquadex Mode	
SCUF	40 (76.9)
CVVH	12 (23.1)
Vascular Access	
Right internal jugular	32 (61.6)
Left internal jugular	5 (9.6)
Right femoral	4 (7.7)
Left femoral	5 (9.6)
Left femoral PICC	1 (1.9)
Right internal jugular and right chest broviac	1 (1.9)
Left internal jugular and left femoral	1 (1.9)
Left femoral and right chest broviac	2 (3.8)
Right chest broviac	1 (1.9)
Catheter size	
5 Fr	1 (1.9)
6 Fr	1 (1.9)
7 Fr	7 (13.5)
7.5 Fr	9 (17.3)
8 Fr	8 (15.4)
9 Fr	6 (11.5)
10 Fr	7 (13.5)
11 Fr	5 (9.6)
11.5 Fr	2 (3.8)
12 Fr	1 (1.9)
13 Fr	5 (9.6)
Blood Flow Rate, mL/kg/min	2.1 (1.1–4.1)

SCUF, slow continuous ultrafiltration; CVVH, continuous veno-venous hemofiltration; PICC, peripherally inserted venous catheter.

**Figure 1:**
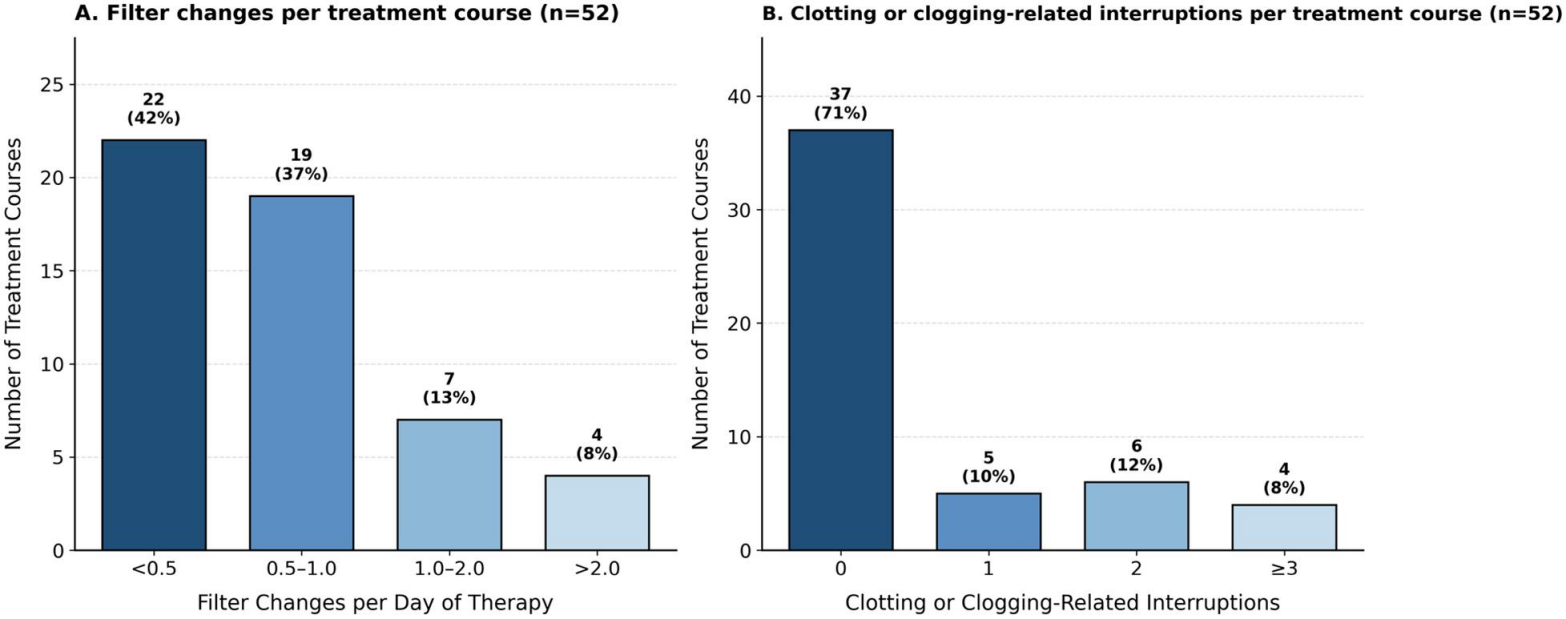
Circuit performance per treatment course. Bars show the number of treatment courses falling into each category, out of 52 total courses in 46 patients. A treatment course was defined as a continuous period of Aquadex therapy interrupted by no more than 24 h; any gap exceeding 24 h was considered the start of a new course (42 patients contributed a single course, 3 patients contributed 2 courses each, and 1 patient contributed 4 courses). **(A)** Distribution of the filter-to-day ratio (the number of filters used per day of therapy), for which lower values indicate longer filter survival. **(B)** Distribution of the number of clotting- or clogging-related interruptions per course, defined as an unplanned cessation of ultrafiltration lasting more than 30 min. Numbers above each bar indicate the count of treatment courses and the corresponding percentage of the cohort.

The median (IQR) duration of Aquadex therapy was 3 (2–9) days, with a median (IQR) of 0 (0–1) interruptions due to circuit clotting or clogging. The median (IQR) ratio of filters used to days of therapy was 0.5 (0.3–0.5). Although the median number of clotting or clogging-related interruptions per course was zero, 15 of 52 courses (29%) experienced at least one such interruption (Figure 1B); among affected courses, the number of interruptions ranged from 1 to 7, with a total of 41 clotting or clogging-related interruptions across the cohort. Circuit performance, expressed as the rate of filter changes per day of therapy, is summarized in Figure 1A. Twenty-two of 52 courses (42%) required fewer than 0.5 filter changes per day (corresponding to an average of more than 48 h per filter), and an additional 19 (37%) required 0.5–1.0 changes per day.

### Platelet count and vasoactive-inotropic score

**Figure 2:**
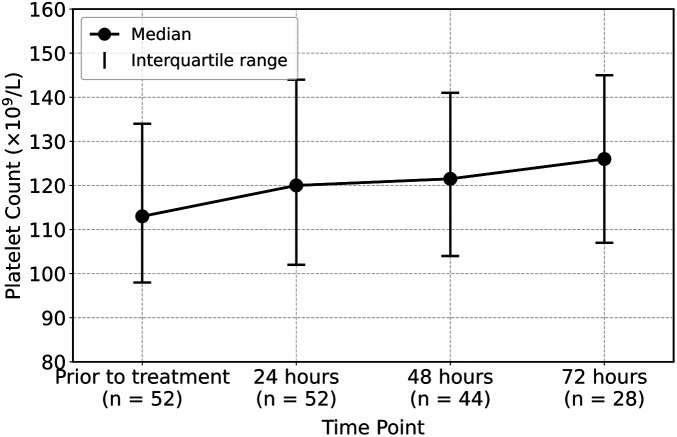
Platelet count during the first 72 h of aquadex therapy. Platelet counts (×10⁹/L) are shown prior to treatment and at 24, 48, and 72 h after the initiation of therapy. Filled circles indicate the median and vertical bars the interquartile range at each time point. Data are derived from 52 treatment courses in 46 patients.

Median platelet counts were higher at each successive time point compared with the immediately preceding measurement: prior to therapy vs. 24 h (*p* < 0.01), 24 vs. 48 h (*p* = 0.02), and 48 vs. 72 h (*p* < 0.01). The median (IQR) number of platelet transfusions administered per day of therapy was 0 (0–1) (Figure 2). No bleeding events directly attributable to epoprostenol use were observed.

**Figure 3:**
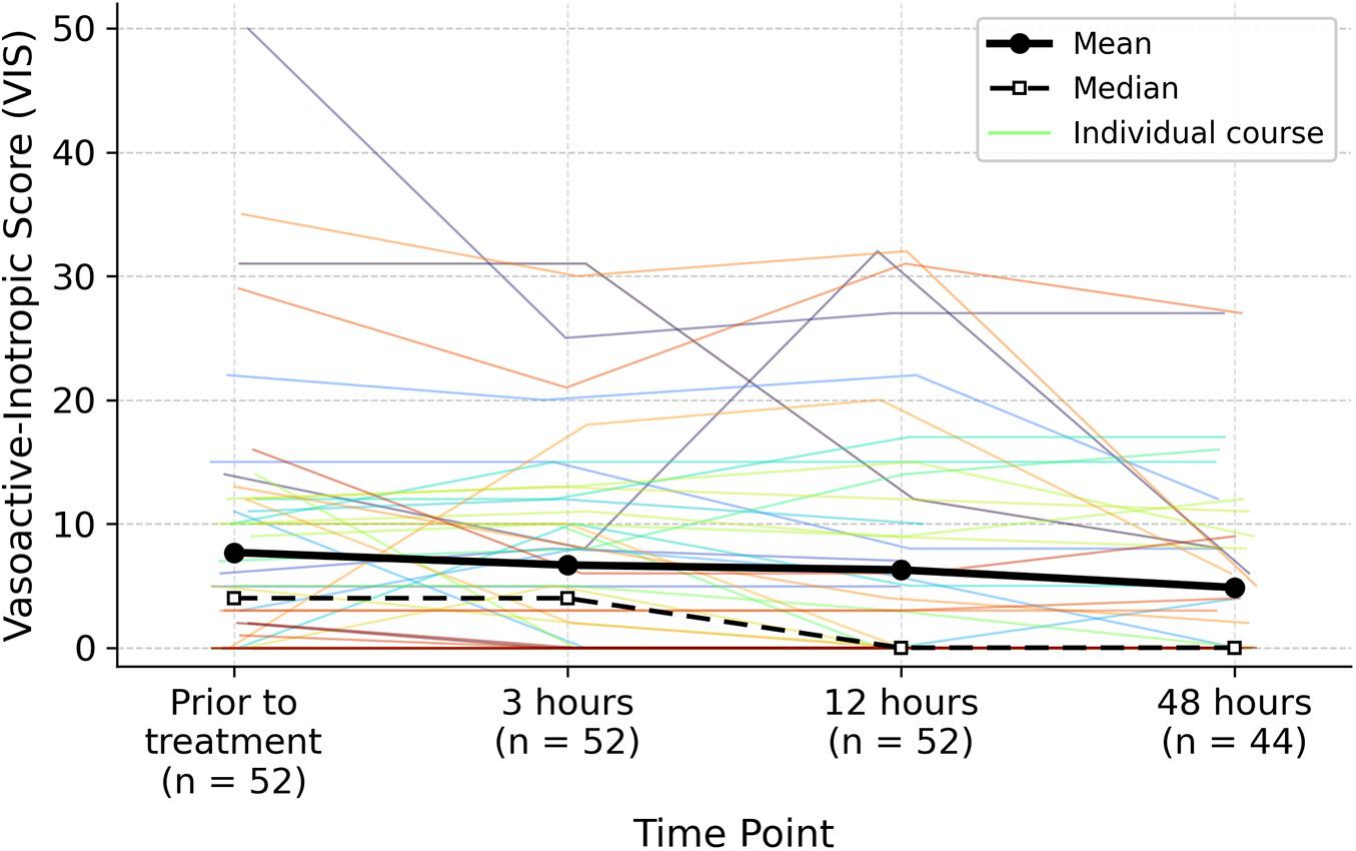
Vasoactive-inotropic score (VIS) over the course of aquadex therapy. Each thin colored line represents a single treatment course (52 courses in 46 patients), with VIS plotted prior to treatment and at 3, 12, and 48 h after the initiation of therapy. The bold black line shows the cohort mean and the dashed black line the median at each time point. The number of courses contributing at each time point is shown in parentheses on the horizontal axis (*n* = 52 prior to treatment, at 3 h, and at 12 h; *n* = 44 at 48 h).

The median (IQR) VIS prior to therapy was 4 (0–12). VIS scores measured at 3, 12, and 48 h after therapy initiation were 4 (0–10.5), 0 (0–9.5), and 0 (0–8), respectively (Figure 3). No statistically significant differences were detected between pre-therapy VIS and any post-initiation time point. No therapy was discontinued because of hypotension.

## Discussion

In this study, we report our experience with the novel use of epoprostenol as an anticoagulant for the Aquadex ultrafiltration system in critically ill pediatric patients. Our findings demonstrate that epoprostenol provided safe and effective anticoagulation, maintaining circuit patency without any significant complications. These results align with prior experience with epoprostenol in continuous renal replacement therapy (CRRT) and highlight its potential use in ultrafiltration systems as a primary anticoagulant or in situations where conventional heparin or citrate-based anticoagulation may not be feasible.

Anticoagulation in extracorporeal therapies remains a significant challenge. Conventional anticoagulants, such as unfractionated heparin or citrate-based anticoagulation, which are most commonly used in clinical practice, may have significant limitations. Unfractionated heparin exhibits variable anticoagulant response due to antithrombin III dependency, carries risk for bleeding, and can lead to heparin-induced thrombocytopenia (HIT) (1, 8). Similarly, citrate-based anticoagulation requires complex calcium and metabolic monitoring and is challenging in patients with impaired citrate metabolism or severe liver dysfunction (12, 13). Epoprostenol, a synthetic analog of prostacyclin, works by inhibiting platelet aggregation through cyclic adenosine monophosphate–mediated pathways and causes local vasodilation without affecting coagulation factors (10). Although prostacyclin inhibits platelet aggregation, its short half-life prevents significant systemic anticoagulant effect. This safety profile aligns with prior reports indicating that prostacyclin does not increase the risk of bleeding in patients with severe coagulopathy or thrombocytopenia (9). Furthermore, prostacyclin does not interfere with coagulation factors or fibrinolysis, differentiating it from unfractionated heparin and supporting its use in critically ill pediatric populations at high risk for bleeding. The short half-life also allows rapid reversal of effect when therapy is paused or completed, avoiding prolonged anticoagulation and eliminating the need for protamine reversal. In contrast to citrate-based anticoagulation, prostacyclin use does not require monitoring of calcium or bicarbonate balance, simplifying bedside management and potentially reducing nursing workload. These operational benefits may be particularly relevant in pediatric intensive care units with limited resources. These characteristics make prostacyclin particularly appealing in situations where both heparin and citrate may be poorly tolerated or contraindicated.

Epoprostenol as an anticoagulant has been investigated in CRRT circuits with proven safety. Deep et al. reported the largest pediatric experience to date. Their study included 96 patients receiving 18,508 h of CRRT with prostacyclin monotherapy, with a median circuit life of 48 h and low bleeding and hypotension rates comparable to those with standard anticoagulants (9). Similarly, other studies in both pediatric and adult populations have found that prostacyclin achieved anticoagulation efficacy and safety comparable to heparin and citrate, with fewer bleeding events in high-risk patients, including those with hepatic dysfunction or severe thrombocytopenia (10, 14).

Our study extends these findings to Aquadex, a low-volume extracorporeal system initially developed for ultrafiltration in adult patients with heart failure but increasingly used in children due to its small extracorporeal circuit and ability to operate at low blood flow rates. Studies have confirmed the safety and feasibility of Aquadex in children, but they utilized heparin as an anticoagulant (1). The use of epoprostenol in our cohort, therefore, represents a novel and clinically significant adaptation of anticoagulation strategy for low-flow extracorporeal therapy.

We did not observe evidence of hemodynamic compromise during therapy. Despite prostacyclin's vasodilatory effects, VIS did not increase during therapy, and no patient required discontinuation due to hypotension. These observations are consistent with prior CRRT studies, where transient hypotension occurred in <5% of sessions and rarely necessitated dose adjustment (9, 10). Importantly, no major bleeding events were attributed to epoprostenol, and platelet counts remained stable.

Our study also demonstrated favorable circuit patency with epoprostenol, with a median filter-to-day ratio of 0.5 and a median of zero clotting or clogging-related interruptions per course, although 29% of courses experienced at least one such interruption. Although direct comparisons with heparin or citrate were not performed, the observed filter performance is consistent with published data using standard anticoagulants in pediatric CRRT and Aquadex therapy (12, 13). Given that filter life is a critical determinant of therapy efficiency and resource utilization, these findings suggest that epoprostenol offers reliable circuit anticoagulation even under low-flow conditions.

This study has several limitations that warrant consideration. Its retrospective, single-center design limits generalizability, and the relatively small sample size restricts statistical power. The absence of a concurrent control group precludes direct comparison of epoprostenol with heparin or citrate anticoagulation. Given the retrospective design, the absence of a comparator group, and the concurrent administration of platelet transfusions and clinical recovery from the precipitating illness, the temporal trend in platelet values should be interpreted as descriptive only and cannot be causally attributed to epoprostenol. Additionally, the lack of standardized dose-response data for epoprostenol titration and the absence of long-term follow-up data are important gaps. Because some patients contributed more than one treatment course, the descriptive analyses treat individual courses as independent observations and do not account for within-patient clustering. Sensitivity analyses restricted to the first course per patient, or mixed-effects models that explicitly account for repeated measures, would be appropriate in larger cohorts. A prospective, multicenter cohort enrolling consecutive eligible patients would improve generalizability and statistical power. Longer-term follow-up capturing thrombotic complications, kidney recovery, and major adverse kidney events would clarify the downstream clinical implications of anticoagulant choice.

## Conclusions

In this first report of prostacyclin use for anticoagulation in Aquadex circuits, epoprostenol demonstrated safe and effective anticoagulation in critically ill pediatric patients. Circuit patency was well maintained, vasoactive-inotropic requirements did not increase, and no bleeding events attributable to epoprostenol were observed. These findings, consistent with prior pediatric CRRT data, support prostacyclin as a viable alternative anticoagulant for low-flow extracorporeal therapies, particularly when heparin or citrate is contraindicated.

## Data Availability

The raw data supporting the conclusions of this article will be made available by the authors, without undue reservation.
